# Erfahrungen mit kombinierten digitalen Lernhilfen bei Datenbank-Vorlesungen

**DOI:** 10.1007/s13222-021-00370-2

**Published:** 2021-03-15

**Authors:** Richard Lenz, David Haller, Dominik Probst, Andreas Wahl

**Affiliations:** 1 Professur für Evolutionäres Datenmanagement, FAU, Martensstrasse 3, 91058 Erlangen, Deutschland; 2 Lehrstuhl für Informatik 6 (Datenmanagement), FAU, Martensstrasse 3, 91058 Erlangen, Deutschland; 3 CS DS MAC-DOP, SIEMENS Mobility, Siemenspromenade 7, 91052 Erlangen, Deutschland

**Keywords:** Blended Learning, Datenbanken, Selbsttest, E‑Assessment, Informatikdidaktik, Probeklausur, Kombinatorische Kontrollfragen, Generierbare Fragen

## Abstract

Im vorliegenden Beitrag berichten wir über die Erfahrungen mit digitalen Lernhilfen und Selbsttests zur Verständnisprüfung am Beispiel einer einführenden Datenbankvorlesung mit weitgehend traditionellen Lehrinhalten. Basierend auf den Rückmeldungen der Studierenden wurde das Angebot an kombinierten digitalen Lernhilfen mit Hilfe der Lernplattform StudOn schrittweise erweitert. Um nicht nur auswendig gelerntes Wissen, sondern auch das Verständnis von Modellen und Konzepten mit automatisch korrigierbaren Fragen prüfen zu können, wurden spezielle Fragetypen entwickelt, die im Wesentlichen auf einfachen kombinatorischen Schlussfolgerungen basieren. Zum Selbsttest für SQL wurde zusätzlich eine Browserübung mit integrierter Datenbank und automatischer Korrektur entwickelt. Die Nutzung der automatisierten Selbsttests konnte deutlich gesteigert werden, indem die Selbsttests zur Zulassungsvoraussetzung für eine freiwillige Probeklausur gemacht wurden. In der Folge konnten hohe Durchfallquoten in der Klausur substantiell gesenkt werden. Im Sommersemester 2020 wurde die Vorlesung auf der Basis der bereits verfügbaren digitalen Lernhilfen vollständig online angeboten und durch virtuelle Vorlesungsbesprechungen im Sinne des Flipped Classroom Modells ergänzt. Aus dem breiten Spektrum der angebotenen digitalen Lernhilfen wurden vor allem die automatisierten Selbsttests stark genutzt und sehr gut bewertet. Die Vorlesungsbesprechungen wurden zwar auch gut bewertet, aber vergleichsweise wenig genutzt. Trotz fehlender Präsenzveranstaltungen haben sich die Klausurergebnisse nicht verschlechtert.

## Einleitung

Die Vorlesung *Konzeptionelle Modellierung* ist eine einführende Datenbankvorlesung, wie sie in ähnlicher Form in vielen Informatik Bachelor Studiengängen zum Pflichtprogramm gehört. Die Vorlesung behandelt das Entity-Relationship-Modell (ER-Modell und erweitertes ER-Modell), das relationale Datenmodell, die Abbildung von ER-Modellen auf das Relationale Datenmodell, Normalisierung, relationale Algebra und SQL. Darüber hinaus gibt es noch Kapitel zu UML, XML, multidimensionaler Modellierung und Ontologien. Zu den Präsenzveranstaltungen gehören 2 SWS Vorlesung und 2 SWS Übungen. Die Besonderheit der Vorlesung an der Universität Erlangen-Nürnberg besteht seit dem doppelten Abiturjahrgang im Jahr 2011 darin, dass die Vorlesung bereits im ersten Semester auf dem Programm steht und neben den Studiengängen Informatik und Wirtschaftsinformatik auch noch zahlreiche weitere Studiengänge mit Informatikanteil oder Nebenfach Informatik bedient, von denen auch einige die Vorlesung im Sommersemester vorsehen. Somit wird die Vorlesung in jedem Semester angeboten, was manche Studierende der Informatik auch als willkommene Gelegenheit zur Wiederholung oder zur Entzerrung des ersten Semesters nutzen. Dies hat zu sehr hohen Teilnehmerzahlen geführt (im Wintersemester regelmäßig etwa 500 Klausurteilnehmer, im Sommersemester etwa 250 Klausurteilnehmer) und damit zu der Notwendigkeit, effiziente möglichst automatisch prüfbare Klausuren zu entwickeln. Das Ziel dabei war, nicht nur auswendig gelerntes Wissen zu prüfen, sondern vor allem auch das Verständnis von Modellen und Konzepten überprüfen zu können. Dazu wurden insbesondere verschiedene Typen kombinatorischer Verständnisfragen entwickelt, bei denen es darum geht, Kardinalitäten von Mengen abzuschätzen. Seit dem Wintersemester 2014 besteht die Klausur zum großen Teil aus automatisch korrigierbaren Multiple-Choice-Fragen, die auch kombinatorische Verständnisfragen zu verschiedenen Themen umfassen. Zusätzlich gibt es immer zur Überprüfung der SQL-Kenntnisse auch einige Fragen zur freitextlichen Ausformulierung von SQL-Anfragen, die natürlich manuell zu korrigieren sind.

Trotz guter Bewertungen in der regelmäßigen Vorlesungsevaluation hatte die Klausur leider anhaltend hohe Durchfallquoten im Bereich über 60 %. Die Ursachen für diese hohen Durchfallquoten sind sicherlich vielfältig. Der Schwierigkeitsgrad der Klausur spielt natürlich auch immer eine Rolle, es sind allerdings auch bei jeder Klausur immer hervorragende Ergebnisse und sehr gute Noten dabei gewesen. Da die Aufgaben in jeder Klausur neu gestellt werden, und die Schwerpunkte jeweils geringfügig variiert werden, können auch Schwankungen im Schwierigkeitsgrad nicht ausgeschlossen werden. Die vielfachen Rückmeldungen der Studierenden aus den Vorlesungsevaluationen legen allerdings nahe, dass die wesentliche Ursache für die hohen Durchfallquoten wohl der zunehmend hohe Lernaufwand der Studierenden im ersten Semester ist. Diese führt dazu, dass Studierende oftmals vermeintlich leichteren Fächern weniger Aufmerksamkeit widmen, um sich besser auf die großen Hürden konzentrieren zu können. Daraus resultiert dann eine mangelnde Kontinuität im Verlauf des Semesters, die auch an den typischen Zugriffszahlen der online bereitgestellten Vorlesungsunterlagen nachvollzogen werden kann: Die Zahl der Studierenden, welche die Vorlesungsunterlagen zeitnah herunterladen, nimmt im Verlauf des Semesters deutlich ab und wenige Wochen vor dem Klausurtermin legt sie sprunghaft zu. In den Klausuren zeigt sich dann regelmäßig, dass scheinbar einfache Konzepte, wie etwa die Semantik von ER-Diagrammen, nicht wirklich verstanden wurden.

Im vorliegenden Beitrag berichten wir über die kombinierten Maßnahmen und „Blended Learning“ Angebote, die entwickelt und evaluiert wurden, um den Studierenden eine gezielte Vorbereitung auf die Klausur zu ermöglichen und eigene Verständnislücken frühzeitig zu erkennen. Insbesondere wurden Selbsttests mit flexibel generierbaren kombinatorischen Kontrollfragen entwickelt, wie sie auch in der Klausur vorkommen. Diese Selbsttests, sowie auch alle anderen hier vorgestellten digitalen Zusatzangebote, sind freiwillig und können von den Studierenden nach Bedarf und Vorliebe genutzt werden. Es hat sich herausgestellt, dass trotz der Bereitstellung vielfältiger Lernhilfen die Durchfallquoten nicht substantiell gesenkt werden konnten, weil es nicht genügend Anreize zur zielorientierten und effektiven Nutzung dieser Lernhilfen gab. Im Wintersemester 2018 wurde dann erstmals eine freiwillige Probeklausur unter echten Klausurbedingungen angeboten. Als deutlich wurde, dass sehr viele Studierende an dieser Probeklausur teilnehmen wollen, wurde das Bestehen der Selbsttests zur Teilnahmevoraussetzung gemacht. Dies hat schließlich zur stark erhöhten Nutzung der Selbsttests und zur deutlichen Reduktion der Durchfallquoten geführt. Über den Effekt der Probeklausur als Anreiz zur Nutzung von digitalen Zusatzangeboten haben wir in [12] berichtet. Als Nebeneffekt der verstärkten Nutzung der Selbsttests war es uns nun auch möglich, das nunmehr messbare Lernverhalten der Studierenden zum Ergebnis der Klausur in Bezug zu setzen.

Im Sommersemester 2020 wurde die Vorlesung aufgrund der Einschränkungen für Präsenzveranstaltungen im Zuge der Covid-19 Pandemie in einem veränderten Modus ausgetragen. Die Vorlesung wurde als Aufzeichnung bereitgestellt, zum regulären Vorlesungstermin wurden zusätzlich Besprechungen als Videokonferenzen angeboten („Flipped Classroom“). Außerdem standen alle bereits entwickelten digitalen Zusatzangebote wie immer zur Verfügung. Erstmals wurde auch ein zusätzlicher SQL-Selbsttest mit frei formulierbarem SQL und direktem Datenbankzugriff angeboten. Am Ende des Semesters wurde eine Befragung der Studierenden durchgeführt, die aufzeigen sollte, welche der vielen digitalen Angebote am meisten genutzt wurden und welche als effektive Lernhilfen wahrgenommen wurden.

## Verwandte Arbeiten

E‑Learning basierte Werkzeuge zur Unterstützung der Datenbanklehre werden bereits seit vielen Jahren an verschiedenen Hochschulen in Deutschland entwickelt und eingesetzt. Thomas Rakow et al. vergleichen in [16] fünf verschiedene Ansätze, die ein breites Spektrum an Funktionen abdecken. Dazu gehören die Lernumgebungen xlx${}^{2}$ der Universität Münster, edb der TH Köln [6] sowie SQLcoach der Hochschule Kaiserslautern [5]. Darüber hinaus gibt es weitere funktional spezialisierte E‑Learning Ansätze, wie das Web-Tool RelaX der Universität Innsbruck, das neben SQL auch Anfragen in der relationalen Algebra unterstützt, oder Werkzeuge für UML-Klassendiagramme [19] sowie für die Normalisierung von Tabellen auf der Basis funktionaler Abhängigkeiten [18].

Einen Schwerpunkt bilden die Lernumgebungen, die den Studierenden mit möglichst geringem Installationsaufwand eine Ausführungsumgebung für das Einüben von SQL-Anfragen bereitstellen. Neben den bereits erwähnten Umgebungen gibt es dafür zahlreiche weitere Werkzeuge (z. B. [3], [17]), die nicht nur eine Trainingsumgebung bereitstellen, sondern auch Feedback zur Korrektheit von SQL-Anfragen liefern können. Eine besondere Schwierigkeit besteht darin, auch für nicht korrekte SQL-Anweisungen automatisch Bewertungen zu generieren, die auch partielle Korrektheit angemessen honorieren. Auch dazu gibt es bereits seit einiger Zeit Vorschläge [10] und auch aktuelle Forschungsansätze [1]. Zhengjie Miao et al stellen mit I‑Rex ein System vor, das eine interaktive Analyse der Auswertungsschritte komplexer SQL-Anfragen ermöglicht, was nicht nur in der Lehre sinnvolle Anwendungen finden dürfte [13].

Einige Lernumgebungen für Datenbankthemen bieten darüber hinaus zusätzliche Funktionen wie die Möglichkeit, Wissen mit Multiple-Choice Aufgaben abzufragen oder die asynchrone Kommunikation mit Studierenden über Foren und Chats. Um dem Effekt des Auswendiglernens entgegenzuwirken, wären Selbsttests mit flexibel generierbaren und automatisiert prüfbaren Fragen wünschenswert. Diesbezüglich wurde bislang jedoch wenig publiziert.

Untersuchungen zu den Bedingungen, zu denen solche Werkzeuge im Rahmen von Blended Learning Veranstaltungen den Lernerfolg verbessern, zeigen bislang kein klares Bild [11]. Speziell für die Angebote zum Thema Datenbanken gibt es zwar Berichte über positive Rückmeldungen von Studierenden, aber ob sich durch die Nutzung der Werkzeuge auch das Stoffverständnis und die Klausurergebnisse verbessern, ist wenig untersucht. Thomas Rakow zeigt in [16] anhand der Zugriffsstatistik, dass die Datenbank-Lernplattform edb von den Studierenden kontinuierlich gut genutzt wird, wobei vor den Klausurterminen jeweils ein Peak auftritt. Das zeigt zwar, dass das Werkzeug besonders zur Klausurvorbereitung durchaus beliebt ist, es ist aber nicht ersichtlich, ob die Studierenden, welche die Online-Hilfen nutzen, in den Klausuren auch bessere Ergebnisse erzielen.

Eine besondere Form des Blended Learning ist das Modell „Flipped Classroom“ oder ICM (Inverted Classroom Model), das beispielsweise von Jens Dittrich an der Universität Saarbrücken erfolgreich für Datenbankvorlesungen umgesetzt wurde [2]. ICM sieht vor, dass die Studierenden sich den Stoff einer Lehreinheit mittels mehrerer kurzer Videoaufzeichnungen bereits im Vorfeld einer Präsenzveranstaltung aneignen. In der Präsenzveranstaltung können dann gemeinsam Aufgaben gelöst werden und der Dozent kann besser auf individuelle Probleme eingehen. Für Vorlesungen wie „*Konzeptionelle Modellierung*“ an der FAU mit sehr vielen Teilnehmern, ist das Modell etwas problematisch, da der gewünschte Effekt der individuellen Problemlösungshilfe schwer erreichbar ist. Studien zur Effektivität des Ansatzes zeigen auch, dass Studierende häufig wenig motiviert und zu undiszipliniert sind, die Videos tatsächlich vor dem Präsenztermin zu erarbeiten [9]. Jüngste Studien legen nahe, dass vor allem Studierende mit hoher Selbstregulierungskompetenz von ICM profitieren [15]. ICM scheint somit kein geeignetes Instrument zu sein, um die Lernkontinuität zu erhöhen.

Als skalierbare Alternative zum Blended Learning können sogenannte MOOCs (Massive Open Online Courses) angesehen werden. Dies sind Lehrangebote, die vollständig dezentral und ohne Präsenzkomponente bereitgestellt werden und automatisierte Leistungsüberprüfungen beinhalten und somit sehr gut auch für über 100.000 Studierende skalieren. Spätestens seit 2011 mit der Online Vorlesung der Stanford Universität von Jennifer Widom werden MOOCs auch in sehr hoher Qualität für das Fach Datenbanken angeboten [14]. Für die universitäre Lehre mit überschaubaren Zahlen von Studierenden scheint ein vollständiger Verzicht auf Präsenztermine im Hinblick auf die Qualität des Lernergebnisses jedoch nicht sinnvoll zu sein [16][7].

## Kombinierte Maßnahmen zum Blended Learning

Die Vorlesung *Konzeptionelle Modellierung* ist in systematisch aufeinander aufbauende Kapitel untergliedert, wobei jedes Kapitel für das Verständnis der nachfolgenden Kapitel wichtig ist. Die Kapitel sind auf die Präsenztermine in den Vorlesungen abgestimmt. Zeitlich versetzt gibt es zu jedem Kapitel in der darauf folgenden Woche eine Übungsaufgabe, die wiederum in der darauf folgenden Woche in den Übungsgruppen besprochen wird. Soweit entspricht die Vorlesung der traditionellen und etablierten Struktur. Zur Ergänzung der Präsenzveranstaltungen werden auch immer noch Sprechstunden beim Dozenten und bei den Tutoren angeboten.

Um die Studierenden bei der Vor- und Nachbereitung sowie bei der Vertiefung und Einübung des Vorlesungsstoffes zu unterstützen, wurde ein breites Spektrum an Blended Learning Angeboten erstellt, die über die Lernplattform StudOn (Studium Online) verfügbar gemacht werden. StudOn ist eine FAU-spezifische Adaption des sehr weit verbreiteten Open Source Learning Management Systems ILIAS [8]. Die hier vorgestellten Erweiterungen sind daher grundsätzlich portierbar und für einen breiten Nutzerkreis wiederverwendbar. Das Angebot wurde nach und nach erweitert und kontinuierlich evaluiert. Ein großer Vorteil der Integration der digitalen Lernhilfen in die Lernplattform ist zum einen der einheitliche Zugang zu einem breit gefächerten digitalen Zusatzangebot, und zum anderen die Möglichkeit der Kopplung verschiedener Angebote, sowie die Möglichkeit zur individuellen Quantifizierung des Lernfortschritts basierend auf verschiedenen Zielkriterien.

Ein wesentliches Ziel der digitalen Angebote ist es, den Studierenden Anreize zu verschaffen, sich mit den zu vermittelnden Inhalten wirklich auseinanderzusetzen, sodass ein vertieftes Verständnis entstehen kann. Dabei geht es nicht nur um eine bloße Präsentation der Inhalte, sondern um die Bereitstellung einer Lernumgebung mit einer guten Mischung aus kognitiven und sozialen Komponenten, welche einerseits zielgerichtet Denkanstöße vermittelt und andererseits bei Verständnisproblemen auch verschiedene Kommunikationskanäle unterstützt (vgl. auch [7]). Dazu wurde für die Vorlesung *Konzeptionelle Modellierung* ein breites Spektrum an digitalen Lernhilfen entwickelt. Eine Übersicht zu allen digitalen Zusatzangeboten findet sich in Tab. 1. Hier wird auch die Chronologie der Erweiterung des digitalen Angebots dargestellt.

Die meisten Angebote wurden auf der Basis der studentischen Rückmeldungen aus der regelmäßigen Vorlesungsevaluation entwickelt. So kam zunächst nach der Umstellung der Klausuren auf viele Multiple-Choice Aufgaben der Kritikpunkt auf, dass die Klausurfragen sich zu sehr von den Fallbeispielen in den Übungen unterscheiden. Um dem zu begegnen, wurden für jedes Kapitel jeweils Kontrollfragen und ein kurzer Selbsttest mit klausurähnlichen Fragen entwickelt. Die Kontrollfragen werden mit den Vorlesungsunterlagen zu jedem Kapitel an die Studierenden ausgegeben. Die zugehörigen Musterlösungen werden in der jeweils darauffolgenden Woche verteilt. Insbesondere wurde versucht, mit den Fragen typische Fehler zu adressieren, die in den Klausuren häufig gemacht wurden.

**Table 1:** 

*Verfügbarkeit*	*Blended Learning Angebot*
Sommersemester 2014 und früher	Alle in der Vorlesung gezeigten Präsentationen (1 Woche vor der Vorlesung)
	Alle Übungsaufgaben (nach der zugehörigen Vorlesung)
	Musterlösungen zu den Übungen (nach der jeweiligen Übung)
	Merkblätter mit Übersichten und zusätzlichen Erklärungen zu schwierigen Themen
	(mit den zugehörigen Vorlesungsunterlagen)
	**Online-Quiz zum Selbsttest** (Jedes Online-Quiz wird nach der zugehörigen Präsenzveranstaltung zur Bearbeitung freigeschaltet)
	Videoaufzeichnung der Vorlesung (Meist kurz nach dem Präsenztermin)
	Zugang zu einer Übungsdatenbank, zur Einübung von SQL (ständig)
	Online-Forum für offene Fragen, die von jedem Kursteilnehmer beantwortet werden können. Bei Bedarf werden Fragen von Tutoren oder dem Dozenten beantwortet. (ständig)
Wintersemester 2015	**Kontrollfragen** zu jedem Kapitel (Bereitstellung mit den Vorlesungsunterlagen)
	Antworten zu den Kontrollfragen (1 Woche nach der Vorlesung)
	Ausgewählte Klausuraufgaben zusammen mit den zugehörigen Musterlösungen und Erläuterungen. Dabei werden vor allem diejenigen Aufgaben erklärt, die in den Klausuren der letzten Jahre besonders schlecht ausgefallen sind.
Sommersemester 2016	Kontrollfragen pro Kapitel mit zusätzlichen **kombinatorischen Verständnisfragen**
	(Bereitstellung mit den Vorlesungsunterlagen)
	Antworten zu den Kontrollfragen (1 Woche nach der Vorlesung)
Wintersemester 2018	**Online-Quiz ergänzt um z. T. automatisch generierte kombinatorische Verständnisfragen**
	**Zulassung zur Probeklausur nur in Verbindung mit bestandenen Online-Selbsttests.**
Wintersemester 2020	**Flipped Classroom Besprechung als Online-Video-Konferenz**
	**SQL-Browserübung mit Datenbankzugriff** **Fragenpool mit unterschiedlichen Schwierigkeitsgraden**

Während anfangs vor allem kurze Wissensfragen in Form von Multiple-Choice Fragen und Zuordnungsfragen in den Kontrollfragen dominierten, wurden nach und nach immer mehr Fragen aufgenommen, deren Beantwortung ein grundlegendes Verständnis voraussetzt. Ziel dabei war es, auch Fragen zu stellen, die zur vertieften Auseinandersetzung mit dem Vorlesungsstoff anregen sollen. Die konstruktive Auseinandersetzung mit dem Vorlesungsstoff ist natürlich auch Gegenstand der regulären Übungen. In den Übungen werden aber eher umfangreiche praxisnahe Fallbeispiele behandelt. Die Kontrollfragen sollen dagegen kurz und prägnant sein, und ganz gezielt typische Verständnisprobleme adressieren.

Die automatisierten Selbsttests enthalten ähnlich kurze prägnante Fragen wie die Kontrollfragen. Sie werden ebenfalls kapitelweise mit jedem Vorlesungstermin freigeschaltet. Sie sind so konfiguriert, dass sie beliebig oft wiederholt werden können. Als bestanden gilt ein Test aber nur, wenn im letzten Durchlauf mindestens 80 % der möglichen Punkte erreicht wurden. Seit dem Wintersemester 2018 enthält der Selbsttest auch generierbare kombinatorische Verständnisfragen, sodass bei wiederholten Testdurchläufen nicht mehr genau die gleichen Fragen gestellt werden.

Das Thema SQL wurde in den Selbsttests bis zum Wintersemester 2019 nur durch MC-Fragen und kombinatorische Fragen abgedeckt. Zum Einüben von SQL wurde eine Übungsdatenbank zur Verfügung gestellt, die von den Studierenden leicht auf dem eigenen Rechner zu installieren ist. Damit findet die Nutzung allerdings außerhalb der Lernplattform statt, was offenbar für viele Studierende bereits eine Hürde zu sein scheint, da die entsprechenden Dateien von vielen Studierenden erst sehr spät oder gar nicht heruntergeladen wurden. Wie diese Datenbank genutzt wurde, ist zudem außerhalb unserer Kontrolle, sodass zu diesem Zusatzangebot kaum Aussagen über den Umfang und die Art der tatsächlichen Nutzung sowie den Beitrag zum Lernerfolg gemacht werden können. Ein weiterer Nachteil der Übungsdatenbank im Vergleich zu den Selbsttests ist, dass es außer der Ausgabe und den Fehlermeldungen der Datenbank keine automatische Korrektur und Bewertung gibt. Um diese Lücke zu schließen, gibt es seit dem Sommersemester 2020 zusätzlich zur Übungsdatenbank eine in StudOn integrierte SQL-Browserübung. Diese ist wie der Selbsttest innerhalb der Lernplattform StudOn implementiert und ermöglicht einen SQL-Selbsttest mit klausurähnlichen Aufgaben, die auf einer integrierten Datenbank auf der Basis von sql.js ausprobiert werden können. Die Studierenden sind damit innerhalb ihrer gewohnten Lernplattform und haben keinerlei zusätzliche Einstiegshürden für den Datenbankzugang. Bei jedem Trainingsdurchlauf können sie bestimmen, ob sie leichte, mittlere oder schwierige Aufgaben bekommen wollen. Die Aufgaben werden dann jeweils aus einem Pool vorformulierter Fragen ausgewählt. Derzeit ist ein Durchlauf so konzipiert, dass je nach Schwierigkeitsgrad etwa 8 bis 12 Fragen ausgewählt werden, wobei neben SELECT Anfragen, sondern auch UPDATE, DELETE, CREATE TABLE und ALTER TABLE geprüft werden. Die direkte Interaktion mit der Datenbank ermöglicht ein unmittelbares Feedback. Ein zusätzliches Feedback zur Korrektheit und Bewertung der SQL-Anweisungen wird derzeit am Ende eines Durchlaufs auf Basis einer quantitativen Analyse verschiedener Kriterien angezeigt. Inwiefern diese bislang recht einfachen Rückmeldungen den Studierenden bei der Korrektur ihrer Anfragen helfen, ist noch zu untersuchen. Die Studierenden können einen Durchlauf unterbrechen und zu beliebigen Zeiten fortsetzen. Ob dieser Interaktionsmodus zielführend zur Verbesserung des SQL-Verständnisses ist, ist ebenfalls noch zu untersuchen.

Bevor die Effektivität des gemischten digitalen Zusatzangebots diskutiert wird, wird im nachfolgenden Abschnitt am Beispiel von ER-Diagrammen, dem relationalen Datenmodell und der relationalen Algebra das Prinzip der kombinatorischen Verständnisfragen erläutert, die leicht automatisch für einen Selbsttest generiert und bewertet werden können.

## Generierbare kombinatorische Verständnisfragen

ER-Diagramme dienen der implementierungsunabhängigen Modellierung von Sachverhalten der realen Welt, die in einer Datenbank abgebildet werden sollen. In verschiedenen Lehrbüchern werden dazu unterschiedliche Notationen verwendet. In der Vorlesung *Konzeptionelle Modellierung* und in den nachfolgenden Darstellungen beziehen wir uns auf das Lehrbuch von Elmasri/Navathe [4]. In typischen Übungsaufgaben wird das Verständnis für die Modellierungsmethode i. A. überprüft, indem eine Situation freitextlich beschrieben wird. Studierende sollen dazu ein geeignetes Diagramm erstellen. Solche Aufgaben mit verschiedenen möglichst realistischen Szenarien sind natürlich notwendig, um die praxisnahe Anwendung der Methode einzuüben, sind aber zeitaufwendig zu bearbeiten, sodass in der verfügbaren Zeit nicht sehr viele solcher Szenarien durchdiskutiert werden können. Lernumgebungen wie edb [16] halten statt der Szenarien Fragenkataloge mit kurzen vorformulierten Texten vor, die jeweils auf einen geeigneten binären Beziehungstyp abzubilden sind. Für die Verständnisprüfung hat diese Methode aber auch Nachteile. Freitextformulierungen können missverständlich sein. Missverständnisse können beispielsweise entstehen, wenn der Text ein Realweltszenario beschreibt, das manche Studierende anders kennen, als im Text beschrieben. Ein weiterer Nachteil ist, dass Werkzeuge wie edb nur leicht zu verstehende binäre Beziehungstypen abfragen. Verständnisprobleme treten aber erfahrungsgemäß vor allem bei mehrstelligen Beziehungstypen auf. Für die Kontrollfragen und automatisierten Selbsttests wurden daher Aufgabenformate entwickelt, welche die automatische Generierung von zufälligen Varianten erlauben. Diese Fragen wurden bewusst so konzipiert, dass die Studierenden dazu gezwungen werden, sich mit der Natur des Modellierungskonstrukts auseinanderzusetzen, unabhängig von Anwendungsbeispielen. Dazu wird die Fähigkeit zu einfachen kombinatorischen Schlussfolgerungen vorausgesetzt. Die Kombinatorik befasst sich mit der Berechnung der Anzahl möglicher Objekte. Es werden also immer Fragen gestellt, bei denen die Antwort nur ein numerischer Wert ist. Wie dies zur Verständnisprüfung im Datenbankbereich genutzt werden kann, soll hier am Beispiel ternärer Beziehungstypen in ER-Diagrammen mit Chen-Notation erläutert werden.

**Figure 1:**
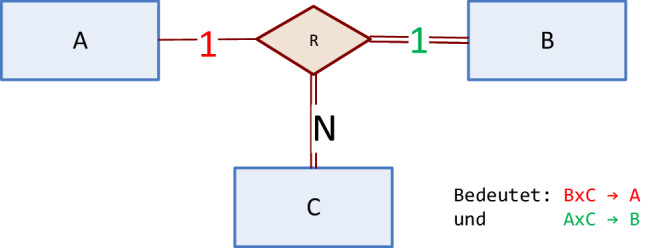

Abb. 1 zeigt einen ternären Beziehungstyp R zwischen drei Entitytypen A, B und C. Jede Ausprägung dieses Beziehungstyps ist ein Tripel $(a,b,c)\in A\times B\times C$. Durch die Angabe von Kardinalitäten (Beschriftung der Kanten mit „1“ oder „N“) werden nun Einschränkungen in der Menge der möglichen Tripel definiert. Die „1“ an der Kante, die A mit R verbindet, definiert eine funktionale Abhängigkeit $B\times C\Rightarrow A$. Das bedeutet, dass eine gegebene Kombination $(b,c)\in B\times C$ höchstens in einem Tripel $(a,b,c)\in A\times B\times C$ vorkommen kann. Analog dazu definiert die „1“ an der Kante zu B eine funktionale Abhängigkeit $A\times C\Rightarrow B$. Die doppelten Linien bei den Entitytypen B und C zeigen deren totale Teilnahme am Beziehungstyp R an: Jedes Entity eines dieser Typen nimmt an mindestens einer Beziehung vom Typ R teil.

Um zu prüfen, ob die Bedeutung dieser Modellierungskonstrukte verstanden wurde, kann man Diagramme wie das in Abb. 1 angeben, zusätzlich die Anzahl der Entities jedes Typs vorgeben und daraus die Zahl der maximal möglichen Beziehungen ausrechnen lassen. Wenn beispielsweise vorgegeben ist, dass es 3 Entities vom Typ A, 4 Entities vom Typ B und 5 Entities vom Typ C gibt, folgt mit $B\times C\Rightarrow A$ dass es nicht mehr als 20 Beziehungen geben kann und mit $A\times C\Rightarrow B$ folgt, dass es nicht mehr als 15 Beziehungen geben kann. Weil beide Bedingungen gelten müssen, ist 15 eine Obergrenze für die Zahl der möglichen Beziehungen vom Typ R. Die totale Teilnahme der Entitytypen B und C spielt für die Obergrenze der Zahl der Beziehungen keine Rolle. Es kann aber alternativ auch gefragt werden: „Wie viele Beziehungen vom Typ R gibt es mindestens?“. Die Untergrenze ist das Maximum der Kardinalitäten der beteiligten Entitytypen mit totaler Teilnahme. Im Beispiel muss es also mindestens 5 Beziehungen von Typ R geben.

Im Selbsttest können solche Fragen mit Variationen automatisch generiert werden, sodass bei wiederholter Ausführung des Tests immer wieder neue Varianten generiert werden. Zu dem Diagramm aus Abb. 1 können beispielsweise leicht die Kardinalitäten der Entitytypen zufällig variiert werden. Aus den Kardinalitäten der Entitytypen (#A, #B, #C) lassen sich dann Unter- und Obergrenze für die Kardinalität des Beziehungstyps #R ausrechnen: $\max(\#B,\#C)\leq\#R\leq\min(\#A*\#C,\#B*\#C)$. Die Variantenvielfalt zum selben Sachverhalt kann weiter erhöht werden indem genau solche Aufgaben für unterschiedliche Diagramme mit den zugehörigen Formeln in einem Aufgaben-Pool bereitgestellt werden, aus denen zufällig eine Aufgabe ausgewählt wird. Theoretisch wäre auch eine automatische Generierung zufällig variierter Diagramme denkbar, das erfordert dann allerdings einigen Programmieraufwand zur korrekten Erzeugung der Grafiken. Die zuvor beschriebene Variantenbildung ist bereits ohne nennenswerten Programmieraufwand allein mit den Konfigurationsmöglichkeiten der Lernplattform StudOn umsetzbar.

Ziel dieser Variantengenerierung ist es, dem Effekt des Auswendiglernens entgegenzuwirken. Dabei ist nur darauf zu achten, dass die jeweilige Zahl der Entities eines Typs klein, aber größer als 1 ist, damit für die Studierenden kein nennenswerter Rechenaufwand entsteht, und dass die Zahlen für die verschiedenen Entitytypen sich unterscheiden, damit die Antwort eindeutig erkennen lässt, ob der Sachverhalt verstanden wurde. Auch für ER-Diagramme mit Min-Max-Notation lassen sich solche kombinatorischen Fragen generieren. Auf diese Weise können auch die Unterschiede zwischen Chen-Notation und Min-Max-Notation mit generierbaren und automatisch korrigierbaren Fragen geprüft werden. Wenn bei einem Diagramm in Min-Max-Notation bei den Kardinalitäten Ober- und Untergrenzen angegeben werden, ist natürlich bei der zufälligen Generierung der Zahl der Entities darauf zu achten, dass dadurch keine Widersprüche entstehen. Dies lässt sich aber leicht durch entsprechende Regeln sicherstellen.

In ähnlicher Weise lässt sich auch das Verständnis für andere Sachverhalte mit flexibel generierbaren kombinatorischen Verständnisfragen überprüfen. Illustriert sei dies mit einer Beispielaufgabe zur Überprüfung der Kenntnis der Primärschlüsseleigenschaft und der referentiellen Integrität im Relationalen Datenmodell:

*Gegeben seien die folgenden Relationen:***R1(A, B, C), R2(D, E, F[R1])** Mit den Wertebereichen:A $\in$ {1,…,100}, D $\in$ {1,…,10} und E $\in$ {1,…,20} 

Nun wird nach der maximalen Anzahl der Tupel in R1 und R2 gefragt. Aufgrund der Primärschlüsseleigenschaft und der gegebenen Wertebereiche können in R1 nicht mehr als 100 Tupel sein, in R2 können zunächst nicht mehr als $20\times 10=200$ Tupel sein. Der Schwierigkeitsgrad kann erhöht werden, indem für das Fremdschlüsselattribut F[R1] zusätzliche Eigenschaften festgelegt werden (UNIQUE und/oder NOT NULL). Wenn F[R1] in R2 zusätzlich UNIQUE (eindeutig) und NOT NULL (ohne NULL-Werte) ist, können aufgrund der referentiellen Integrität in R2 nicht mehr als 100 Tupel sein.

Solche Aufgaben können analog zu den oben erläuterten ER-Aufgaben zufällig generiert und automatisch geprüft werden, indem Attribute, Attributeigenschaften und Wertebereiche zufällig variiert werden. Nach dem gleichen Prinzip können theoretisch auch komplexere Aufgaben automatisch generiert werden, indem beispielsweise auch noch die Zahl der Tabellen erhöht wird. Es ist aber darauf zu achten, dass dabei nicht Aufgaben entstehen, bei denen zur Lösung nicht mehr das zu prüfende Verständnis im Vordergrund steht, sondern die kombinatorischen Fähigkeiten der Studierenden.

In den Kontrollfragen und Selbsttests zur Vorlesung *Konzeptionelle Modellierung* sind neben traditionellen Fragetypen, wie Multiple-Choice und Zuordnungsfragen, auch viele weitere kombinatorische Verständnisfragen zu anderen Themenbereichen enthalten. Insbesondere zur Relationalen Algebra und SQL lassen sich leicht solche Fragen generieren. Beispielsweise kann zum oben definierten relationalen Schema gefragt werden, wie viele Tupel durch eine Verbundoperation wie beispielsweise $\texttt{JOIN}_{\texttt{R1.A=R2.F}}$ (R1, R2) höchstens geliefert werden können. Die Frage kann nur beantwortet werden, wenn neben der Primärschlüsseleigenschaft und der referentiellen Integrität auch die Semantik der Verbundoperation verstanden ist. Im vorliegenden Beispiel kann der Verbund maximal 200 Tupel enthalten, denn jedes Tupel in der Fremdschlüsselrelation R2 findet höchstens einen Verbundpartner in R1. Die Frage kann variiert werden durch unterschiedliche Wertebereiche der Attribute, unterschiedliche Verbundprädikate oder unterschiedliche Operationen, wie z. B. äußerer Rechtsverbund oder äußerer Linksverbund. Dadurch können durchaus anspruchsvolle Denkaufgaben generiert werden. Einige Variationen für das oben genannte Beispiel:

- $\texttt{RIGHT OUTER JOIN}_{\texttt{R1.A=R2.F}}$ (R1, R2) liefert nach wie vor maximal 200 Tupel, denn nach wie vor gibt es zu jedem Tupel in der Fremdschlüsselrelation R2 maximal ein Tupel im Ergebnis. Das gilt auch dann, wenn F kein Fremdschlüsselattribut ist, und es gilt auch dann, wenn A nicht Primärschlüssel aber UNIQUE ist.
- $\texttt{LEFT OUTER JOIN}_{\texttt{R1.A=R2.F}}$ (R1, R2) liefert maximal 299 Tupel. Dieser Wert wird dann erreicht, wenn alle Tupel in R2 den gleichen Wert im Fremdschlüsselattribut F haben. Dann gibt es nur ein Tupel aus R1, das Verbundpartner für alle Tupel in R2 wird. Die maximal 99 weiteren Tupel aus R1 werden mit NULL-Werten aufgefüllt und dem Ergebnis hinzugefügt.
- $\texttt{JOIN}_{\texttt{R1.B=R2.F}}$ (R1, R2) liefert maximal 20.000 Tupel. Dieser Wert wird erreicht, wenn R1.B $=$ R2.F für alle Tupel im Kreuzprodukt gilt, was hier möglich ist wenn der Attributwert in B und F konstant ist.

Noch ein wenig schwieriger ist beispielsweise bereits die Frage nach der maximalen Anzahl der Tupel in $\texttt{JOIN}_{\texttt{R1.B=R2.E}}$ (R1, R2). Hier können maximal 1.000 Tupel im Ergebnis sein, denn jedes Tupel aus R1 findet maximal 10 Verbundpartner in R2, weil ein bestimmter Attributwert im Attribut E höchstens 10 mal vorkommen kann, damit die Eindeutigkeit des Primärschlüssels $\{D,E\}$ gewährleistet bleibt. Es kann auch keine kleinere Schranke geben, denn 1.000 Tupel werden tatsächlich erreicht, wenn R1 100 Tupel hat, B konstant ist und dieser konstante Wert 10 mal in R2.F vorkommt.

Abschließend sei bemerkt, dass die kombinatorischen Verständnisfragen nicht die Anwendung mit realistischen Beispielen ersetzen können. Sie dienen nur als Anreiz, sich mit den grundlegenden Sachverhalten gedanklich auseinanderzusetzen, die dem Vorlesungsstoff zugrunde liegen, und als komplementäre Methode zur automatisierbaren Verständnisprüfung.

## Evaluation zur Effektivität von gemischten Lehrmethoden

**Figure 2:**
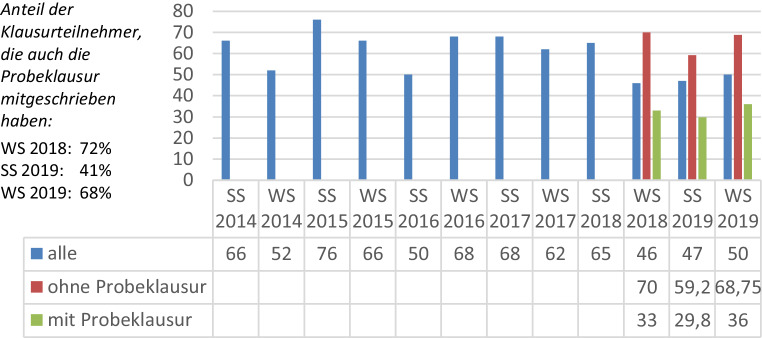

Die besten digitalen Lernhilfen sind nicht effektiv, wenn sie nicht zielorientiert genutzt werden. So wurden die hier beschriebenen digitalen Zusatzangebote bis zum Wintersemester 2018 vergleichsweise wenig genutzt. Erst mit der Kopplung der Selbsttests als Zulassungsvoraussetzung zur freiwilligen Probeklausur konnte ein ausreichender Anreiz geschaffen werden, das Angebot auch während des Semesters kontinuierlich zu nutzen. In Abb. 2 wird der Effekt dieser Maßnahme anhand der Durchfallquoten verdeutlicht (vgl. [12]).

Auch im Sommersemester 2020 konnte der positive Trend fortgesetzt werden, obwohl die Probeklausur diesmal nicht unter Echtbedingungen stattfinden konnte, sondern nur als elektronisches Dokument zum Selbstausdrucken an die Studierenden mit bestandenen Selbsttests verteilt wurde. Die Teilnahme an der Probeklausur war im Sommersemester 2020 etwas niedriger als in den vorangegangenen Semestern. Von 181 Klausurteilnehmern (330 Klausuranmeldungen) haben sich 52 (etwa 30 %) durch regelmäßig bestandene Selbsttests für die Probeklausur qualifiziert und an dieser teilgenommen. Die Teilnahme an den Selbsttests ist im Sommersemester gewöhnlich geringer als im Wintersemester, weil viele Wiederholer aus dem Wintersemester die gleichen Selbsttests und die gleiche Probeklausur nicht noch einmal absolvieren wollen. Es ist nicht unwahrscheinlich, dass der Rückgang der Teilnahme in diesem Semester nicht auch auf die veränderten Bedingungen im Rahmen der Pandemiebeschränkungen und die fehlende Probeklausur unter Echtbedingungen zurückzuführen ist. Aus diesem Grund wurden die Zahlen für das Sommersemester 2020 nicht in Abb. 2 aufgeführt. Die tatsächlichen Durchfallquoten aus dem Sommersemester 2020 sind geringer als in den vorherigen Semestern, aber nicht vergleichbar, weil erstmals ein anderer gesetzlich vorgeschriebener Korrekturmaßstab angewendet wurde. Die vergleichbaren Durchfallquoten nach dem alten Korrekturmaßstab liegen in dem Bereich der Vorsemester und bestätigen damit den positiven Trend. Demnach liegt die Durchfallquote insgesamt bei 47,5 %. Von den Teilnehmern mit Qualifikation für die Probeklausur haben 38,9 % nicht bestanden, von den übrigen sind 50,8 % durchgefallen.

Insgesamt konnten mit der Probeklausur und den damit verbundenen Selbsttests die Durchfallquoten von 60–70 % auf zumindest unter 50 % gesenkt werden. Diese Entwicklung legt die Schlussfolgerung nahe, dass die regelmäßige Teilnahme an den Selbsttests die Klausurergebnisse deutlich verbessern kann. Eine genauere Analyse der personalisierten Daten aus den Selbsttests zeigt jedoch ein etwas differenzierteres Bild. Tatsächlich liegen die Durchfallquoten der Studierenden, die regelmäßig an den Tests teilgenommen haben, mit etwas über 30 % sehr deutlich unter den Durchfallquoten der Studierenden, die nicht regelmäßig teilgenommen haben (60–70 %). Besonders hohe Durchfallquoten wurden aber auch bei den Studierenden festgestellt, die die Selbsttests besonders oft gemacht haben. Manche Studierende haben den selben Test mehr als 10 mal durchlaufen. Gerade diese Studierenden haben ein sehr hohes Risiko bei der Klausur durchzufallen. Zumindest bei den Multiple-Choice Fragen, die nach wie vor im Test vertreten sind, führt der wiederholte Selbsttest offenbar eher zum unreflektierten Auswendiglernen, aber nicht unbedingt zu einem besseren Verständnis. Dieses Ergebnis spricht auch dafür, dass solche automatisierten Selbsttests nicht unbedingt geeignet sind, sich den Stoff anzueignen, sondern eher dazu gedacht sind, die eigenen Verständnislücken aufzudecken. Um die Studierenden nicht in falscher Sicherheit zu wiegen, sollten nach Möglichkeit wiederholte Testdurchläufe zu unterschiedlichen Fragen führen. Dies spricht auch für generierbare Fragen, die in vielen Varianten immer wieder neu erzeugt werden können. Alternativ oder ergänzend dazu können große Fragen-Pools zu verschiedenen Themen entwickelt werden, die inkrementell um immer neue Fragen ergänzt werden, und aus denen dann zufällig Fragen aus definierten Themenbereichen ausgewählt werden. Themenbezogene Fragenpools ermöglichen den Studierenden auch die gezielte Adressierung der eigenen Defizite. Mit der Bereitstellung der SQL-Browserübungen steht den Studierenden an der FAU seit dem Sommersemester 2020 zumindest für SQL ein solcher themenbezogener Fragenpool zur Verfügung.

**Figure 3:**
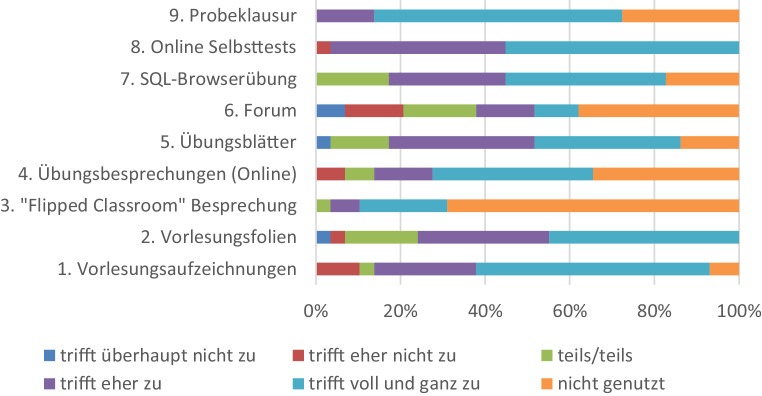

Um festzustellen, welche der vielen digitalen Zusatzangebote besonders effektiv waren, wurde zum Ende des Sommersemesters 2020 zusätzlich zur regulären Vorlesungsevaluation eine studentische Befragung durchgeführt, deren Ergebnisse nachfolgend zusammengefasst werden. Auch wenn die Befragung mit nur 29 Rückläufern bei 181 Klausurteilnehmern vergleichsweise wenig Resonanz fand, und somit auch nicht als repräsentativ angesehen werden kann, so sind doch tendenziell interessante Ergebnisse abzulesen. Die Antworten auf die Frage „Welche Lehrmaterialien haben mir weitergeholfen?“ werden in Abb. 3 zusammengefasst.

Interessant ist hier vor allem die außerordentlich positive Bewertung der Selbsttests, die sogar noch vor der Vorlesungsaufzeichnung und den Vorlesungsfolien rangiert, und die sogar mehr genutzt wurden als die eigentlichen Übungsblätter und Übungsbesprechungen. Die neu eingeführte automatisierte SQL-Browserübung wurde noch nicht so ausgiebig genutzt wie die Selbsttests, findet aber auch überwiegend positive Bewertungen und wurde ebenfalls besser genutzt als die Übungsbesprechungen. Am wenigsten hilfreich war offenbar das Forum, das für den öffentlichen Austausch über ungeklärte Fragen vorgesehen war. Tatsächlich wurde in diesem Semester das Forum vergleichsweise wenig genutzt. Bemerkenswert ist auch die Bewertung der Flipped Classroom Besprechungen: Kein anderes Angebot wurde weniger genutzt, aber die Teilnehmer an den Besprechungen bewerten diese sehr positiv. Schließlich ist die außerordentlich positive Bewertung der Probeklausur selbst hervorzuheben. Das mag damit zusammenhängen, dass die Musterlösung zur Probeklausur nicht nur die Lösungen zu den Aufgaben enthält, sondern auch für jede Lösung eine ausführliche Begründung.

**Figure 4:**
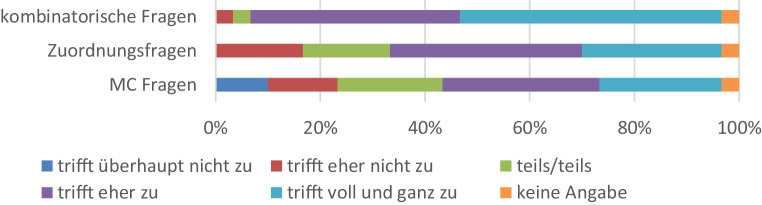

Um die Effektivität der Selbsttests weiter verbessern zu können, wurden in der Befragung auch verschiedene Aufgabentypen bewertet. Dabei wurde zwischen Multiple-Choice-Fragen, Zuordnungsfragen und kombinatorischen Fragen unterschieden. Gefragt wurde inwiefern die verschiedenen Aufgabentypen zum Verständnis des Stoffes beigetragen haben. Die aggregierten Ergebnisse sind in Abb. 4 dargestellt. Es zeigt sich, dass vor allem die kombinatorischen Fragen als sehr hilfreich angesehen wurden.

Die SQL-Browserübung wurde in der Befragung insgesamt positiv bewertet. Ob dadurch aber tatsächlich ein besseres Verständnis für SQL erreicht werden konnte, lässt sich auf der Basis der aktuellen Klausurergebnisse und Rückmeldungen noch nicht bewerten. Die Nutzungsstatistiken der SQL-Browserübung zeigen allerdings bereits ein hohes Interesse an dieser Form der digitalen Lernhilfe, obwohl die Übung erst relativ spät im Semester und nach der zugehörigen Vorlesung freigeschaltet werden konnte. In der Kategorie mit leichten Fragen waren 135 Teilnehmer, von denen 83 bestanden haben. Die Kategorie mit mittelschweren Fragen hatte 110 Teilnehmer (47 bestanden) und die Kategorie mit schwierigen Fragen hatte 91 Teilnehmer (19 bestanden). Viele der Teilnehmer, die nicht bestanden haben, haben sich offenbar auch nur die Fragen angesehen, ohne wirklich eine Bearbeitung zu beginnen.

## Zusammenfassung und Ausblick

Digitale Lernhilfen sollen den Studierenden helfen, sich einerseits zielorientiert mit dem Vorlesungsstoff auseinanderzusetzen, andererseits auch den eigenen Kenntnisstand und vor allem die eigenen Defizite besser einschätzen zu können. Für die einführende Datenbankvorlesung *Konzeptionelle Modellierung* wurde ein breites Spektrum an verschiedenen digitalen Lernhilfen entwickelt, die weitgehend innerhalb der Lernplattform StudOn bereitgestellt werden. Die homogene Lernumgebung ermöglicht die schnelle Bereitstellung neuer Lernhilfen, ohne dass die Studierenden dafür etwas Neues installieren müssen oder sich an eine neue Software gewöhnen müssen. Darüber hinaus fallen durch die Nutzung detaillierte Daten an, die zur Analyse des Lernverhaltens genutzt werden können und eine kontinuierliche zielorientierte Verbesserung des Angebots ermöglichen. Neben der schlichten Bereitstellung von Kommunikationskanälen und Unterlagen verschiedenster Art wurden für die Vorlesung *Konzeptionelle Modellierung* vor allem ein Online-Selbsttest und zuletzt eine SQL-Browserübung mit automatisierter Bewertung bereitgestellt. Durch die bloße Bereitstellung solcher Lernhilfen konnten die Klausurergebnisse allerdings zunächst nicht verbessert werden. Erst als das Bestehen der Online-Selbsttests zur Zulassungsvoraussetzung für eine freiwillige Probeklausur gemacht wurde, konnten die Nutzungsraten der Selbsttests deutlich erhöht werden und die Durchfallquoten in der Klausur wurden gesenkt. Offenbar ist es wichtig, die geeigneten Anreize zu schaffen, damit die Studierenden die Angebote tatsächlich nutzen.

Die vorläufige Auswertung der Nutzung der verschiedenen digitalen Lernhilfen hat ergeben, dass vor allen die automatisierten Selbsttests und die Probeklausur zum Lernerfolg beigetragen haben. Insbesondere die kombinatorischen Fragen scheinen gemäß der studentischen Rückmeldungen zum Verständnis des Stoffes beigetragen zu haben. Diese Fragen haben außerdem den Vorteil, dass sie in einem automatisierten Selbsttest bei wiederholtem Durchlauf immer wieder in verschieden Variationen und mit unterschiedlichen Parametern und verschiedenen Schwierigkeitsgraden generiert werden können. Dies ist umso wichtiger, da die Auswertungen auch gezeigt haben, dass Online-Selbsttests bei sehr häufiger Wiederholung zum Auswendiglernen führen können, anstatt weiter zum Verständnis beizutragen. Um dem entgegenzuwirken, sollten nach Möglichkeit neben der automatischen Generierung von Fragen auch andere Optionen, wie Fragenpools mit vielfältigen Fragetypen ausgenutzt werden.

Da die neu entwickelte SQL-Browserübung erst zum Ende des Sommersemesters 2020 bereitgestellt wurde, können dazu noch keine detaillierten Auswertungen vorliegen. Die Akzeptanz dieses digitalen Zusatzangebots scheint aber sehr hoch zu sein und erste Bewertungen sind auch eher positiv. Ein möglicher Effekt auf die Klausurergebnisse ist nicht messbar, da die Veranstaltung in diesem Semester aufgrund der Pandemiebeschränkungen ohne Präsenzveranstaltungen und damit unter veränderten Bedingungen stattfinden musste. Statt der Präsenzvorlesung wurde zur Vorlesungsaufzeichnung eine Online-Besprechung im Sinne des Flipped Classroom-Modells angeboten. Diese Besprechungen wurden zwar vergleichsweise wenig genutzt (etwa 20-30 Teilnehmer), aber sehr positiv bewertet. Da die Durchfallquoten im Sommersemester 2020 trotz fehlender Präsenzveranstaltungen sogar weiter gesenkt werden konnten, scheint sich das breit gefächerte digitale Lehrangebot insgesamt zu bewähren.

Für die kommenden Semester ist ein weiterer Ausbau der Lernumgebung geplant. Insbesondere sollen die Fragenpools für die Selbsttests erweitert werden, und das Angebot zum zielorientierten SQL-Training soll weiter ausgebaut und verbessert werden. Grundsätzlich ermöglicht die integrierte Lernumgebung auch die Durchführung von E‑Klausuren, die auf der Basis bestehender Fragenpools automatisch erzeugt werden könnten.
